# Bronchodilatory and Anti-Inflammatory Effects of ASM-024, a Nicotinic Receptor Ligand, Developed for the Treatment of Asthma

**DOI:** 10.1371/journal.pone.0086091

**Published:** 2014-01-22

**Authors:** Evelyne Israël Assayag, Marie-Josée Beaulieu, Yvon Cormier

**Affiliations:** 1 Institut Universitaire de Cardiologie et de Pneumologie de Québec, Québec, QC, Canada; 2 Asmacure Ltée, Québec, QC, Canada; University of Rochester Medical Center, United States of America

## Abstract

Conventional asthma and COPD treatments include the use of bronchodilators, mainly β2-adrenergic agonists, muscarinic receptor antagonists and corticosteroids or leukotriene antagonists as anti-inflammatory agents. These active drugs are administered either separately or given as a fixed-dose combination medication into a single inhaler. ASM-024, a homopiperazinium compound, derived from the structural modification of diphenylmethylpiperazinium (DMPP), has been developed to offer an alternative mechanism of action that could provide symptomatic control through combined anti-inflammatory and bronchodilator properties in a single entity. A dose-dependent inhibition of cellular inflammation in bronchoalveolar lavage fluid was observed in ovalbumin-sensitized mice, subsequently treated for 3 days by nose-only exposure with aerosolized ASM-024 at doses up to 3.8 mg/kg (ED_50_ = 0.03 mg/kg). The methacholine ED_250_ values indicated that airway hyperresponsivenness (AHR) to methacholine decreased following ASM-024 administration by inhalation at a dose of 1.5 mg/kg, with a value of 0.145±0.032 mg/kg for ASM 024-treated group as compared to 0.088±0.023 mg/kg for untreated mice. In *in vitro* isometric studies, ASM-024 elicited dose-dependent relaxation of isolated mouse tracheal, human, and dog bronchial preparations contracted with methacholine and guinea pig tracheas contracted with histamine. ASM-024 showed also a dose and time dependant protective effect on methacholine-induced contraction. Overall, with its combined anti-inflammatory, bronchodilating and bronchoprotective properties, ASM-024 may represent a new class of drugs with a novel pharmacological approach that could prove useful for the chronic maintenance treatment of asthma and, possibly, COPD.

## Introduction

Asthma is a respiratory disease characterised by airway inflammation and hyperresponsiveness resulting in reversible bronchoconstriction that affects between 8 to 10% of the population in industrialised countries [1]. Current treatment is mostly based on inhaled corticosteroids and β2 receptor agonists used individually or in combination [2]. Despite these effective treatments, half of asthmatics are not adequately controlled [3]. The reduced efficacy associated with the overuse of β2 agonists leading to β2-adrenergic receptor tachyphylaxis may play role in this inadequate control in chronically-treated asthmatics [4], [5]. Moreover, about 5 to 10% of patients with severe asthma fail to respond to inhaled or oral glucocorticosteroids [6]–[8]. Most importantly, high doses and long term use of inhaled corticosteroids have been associated with significant and sometimes serious side effects [2]. Because of these unmet needs, the development of new treatments for asthma is warranted, especially for drugs that have a different mode of action, thus potentially bypassing the limitations of current medication [9].

ASM-024 is an analogue of dimethylphenylpiperazinium (DMPP), a well-described nicotinic receptor agonist. ASM-024 is currently in Phase II trials for the treatment of asthma and COPD. The pharmacological target, scientific rationale and working hypothesis for the development of ASM-024 were based on the key role of the cholinergic system in the respiratory tract, not only for the control of airway smooth muscle tone, but also in the regulation of inflammation [10]. Nicotinic receptors (nAChRs) are expressed on parasympathetic nerves but also on airway structural, inflammatory and smooth muscle cells support their putative regulatory role in lung inflammatory diseases and particularly asthma [11], [12]. Several studies have demonstrated that acetylcholine is also synthesized by nonneuronal cells including inflammatory and epithelial cells and is involved in the modulation of inflammation through interaction with nicotinic [13], [14] and muscarinic receptors [15]. Epidemiological studies showing a lower incidence of several inflammatory diseases in smokers and anecdotal clinical observations of asthma exacerbations or even *de novo* asthma development after smoking cessation [16], [17] also support this hypothesis. The mechanisms underlying the protective effects of nicotine on the development of these inflammatory diseases may be linked to the well documented nicotine-induced anti-inflammatory cascade [18], [19]. Based on the above, the pharmacological approach targeting nAChRs and subsequent activation of the anti-inflammatory pathway was proposed for the control of inflammation in asthma pathogenesis.

Early investigations demonstrated that DMPP is highly effective in decreasing lung inflammation and lung tissue resistance in a mouse model of allergic airway inflammation and hyperreactivity [20], presents smooth muscle relaxant properties [21] and inhibits eosinophil migration [22]. Following these proofs of concept studies, ASM-024, a small positively charged homopiperazinium compound, was selected for drug development for the treatment of respiratory inflammatory diseases such as asthma. Due to its quaternary ammonium structure, ASM-024 does not penetrate the blood brain barrier thereby limiting any potential for central effects including addiction and/or dependence. In standard radioligand receptor binding competition assays, ASM-024 showed 60% inhibition at 30 µM with an IC_50_ of 19 µM and a K_i_ of 13 µM for nonselective nAChR subtypes, and low binding affinity for most of the nAChR subtypes tested, except for the human α3β4 receptor, for which the K_i_ was 0.88 µM. Recent observations from whole cell voltage clamp experiments have revealed that ASM-024 inhibits acetylcholine-evoked responses on human α3β4 and α7 subtypes expressed in *Xenopus* oocytes, indicating a potential antagonist effect. However, when co-applied with the type II α7 positive allosteric modulator, PNU-120596, ASM-024 appears to function as an agonist and effectively activates the α7 ion channel [23]. Compounds with similar properties are defined as “silent agonists” and were reported to have anti-inflammatory effects [24]. In addition, although a receptor binding assay indicated low binding affinity for the various muscarinic receptor (mAChRs) subtypes, ASM-024 was shown to decrease muscarinic responses to acetylcholine of M1, M2, and M3 mAChR expressed in *Xenopus* oocytes. These observations indicate a complex multifunctional mechanism of action which is still being investigated. The rather recent finding of the effect of ASM-024 at the muscarinic level has led to the effort to explore the potential clinical use of the compound in the treatment of COPD. The objective of this paper is to describe the pharmacological properties of this new molecule. For this purpose we investigated the effects of ASM-024 on inflammatory response and airway smooth muscle contractility and relaxation using *in vivo* and *in vitro* preclinical models.

## Materials and Methods

### ASM-024 Pharmacological Activity

To evaluate the pharmacological effects of ASM-024, studies were conducted *in vivo* with a mouse model of allergic airway inflammation and hyperreactivity and *in vitro* with tracheal or bronchial preparations obtained from mice, guinea pigs, dogs, and humans. This study was carried out in strict accordance with the recommendations in the Guide for the Care and Use of Laboratory Animals of the Canadian Council on Animal Care (CCAC) regarding acclimation, environmental enrichment, and analgesic and anesthesia procedures. The protocols were approved by the Committee on the Ethics of Animal Experiments of Université Laval. Tissues from human donors were obtained after written informed consent from all individuals in accordance with an Internal Review Board-approved protocol at IUCPQ Research Center (Institut universitaire de cardiologie et de pneumologie de Québec) in Québec, QC (Canada).

#### Mouse Model of allergic airway inflammation and hyperreactivity

Balb/c mice (n = 7–9 for each group) were intraperitoneally sensitized with 100 µg/100 µL ovalbumin (OVA) in 2% (w/v) aluminium hydroxide on days 0 and 7. OVA-sensitized mice were intranasally challenged with the allergen OVA (50 µg/50 µL) on days 20–23. On day 24, mice were sacrificed and bronchoalveolar alveolar lavage (BAL) was performed; the number of total cells recovered in the BAL was used as a measure of the severity of lung inflammation. Cells were stained with Hema 3 Stain Set (Fisher Scientific) and differential cell counts were performed by two observers. Lymphocytes were distinguished from macrophages based on shape, coloration and morphology of the cells and nuclei. Appropriate positive and negative controls were done in parallel with the treated groups. Separate groups of OVA-sensitized mice were used for the measure of hyperreactivity.

*Oral Drug Delivery*. OVA-sensitized animals were treated with various doses of ASM-024 dissolved in water (0.03 to 30 mg/kg) by oral gavage (p.o.) of 100 µL on days 21–23, 30 minutes before the OVA intranasal challenge. In some experiments aimed at measuring airway responsiveness (AR), the drug was given for 3 or 7 days p.o. and the AR measured. Control mice were treated with water only.

*Inhalation Drug Delivery*. Mice were sensitized and challenged with OVA as described above. Aerosolized ASM-024 or saline (vehicle control) was administered using the inExpose system, a nose-only aerosol exposure tower from Scientific Respiratory Equipment Inc. (SCIREQ) (Montréal, QC, Canada). Aerosol was delivered with the Aeroneb® Lab Micropump Nebulizer, producing particle size of 1.8 µm MMAD, 2.0 µm GSD. The animals were exposed to different doses of ASM-024 for 25 minutes on days 20–23. OVA challenges were done 30 minutes after the end of exposure of ASM-024 aerosol. Target doses were 0.03, 0.3 and 3 mg/kg. Actual administered doses were calculated according to the following formulas:

Estimation of inhaled volume per mouse 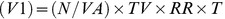


Estimation of ASM-024 administered dose 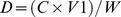


Where N is nebulisation rate, V_A_ is air flow (2 L/min), T_V_ is tidal volume (estimated at 170 µl), R_R_ is respiratory rate (estimated at 150 breath/min), T is the duration of treatment, C is ASM-024 concentration solution and W is mouse weight (18–20 g).

Based on these parameters, the estimated administered doses were, 0.038, 0.38, 3.80 mg/kg.

*In Vivo Airway Responsiveness in Allergen-Sensitized Mice*. On day 24, OVA sensitized and challenged mice were exposed to a single dose of 1.5 mg/kg aerosolized ASM-024, 10 minutes prior the methacholine challenge. AR was measured using the FlexiVent apparatus (SCIREQ, Montréal, QC, Canada). Mice were anesthetised with ketamine-xylazine, tracheotomised and intubated with an 18 G catheter and paralysed with pancuronium (0.5 mg/kg). Respiratory frequency was set at 160 breaths/min, a tidal volume of 0.2 mL and a positive end-expiratory pressure of 3 mL H_2_O. Increasing concentrations of methacholine (0–1 mg/kg) were administered intravenously. The Snapshot perturbation maneuver was imposed to measure airway resistance (R). A lower resistance response to methacholine (R) value for OVA-sensitized and ASM-024-treated compared to untreated mice indicates lower airway responsiveness. Results are expressed as percentage of increase resistance. The effective dose of methacholine (mg/kg) required to induce a 250% (ED_250_) increase of airway resistance was calculated.

#### In vitro Smooth Muscle Relaxant and Bronchoprotective Effects

The effects of ASM-024 on airway smooth muscle were investigated in isometric studies performed on tracheal and bronchial preparations. Tracheas were isolated from naïve mice and guinea pigs. Human bronchi were isolated from healthy lung tissue obtained from patients undergoing resection for lung cancer. Human tissues were obtained from the IUCPQ site of the Respiratory Health Network Tissue Bank of the FRQS (www.tissuebank.ca), subjects were non-smokers or ex-smokers for more than 6 months with normal respiratory functions. Bronchi were also isolated from lung of euthanized healthy dogs.

Tracheal or bronchial preparations devoid of adherent connective tissue were suspended in 5 mL organ baths containing Krebs solution. Krebs composition in mM: NaCl 118, KCl 4.7, KH_2_PO_4_ 1.18, MgSO_4_ 1.18, NaHCO_3_ 25, glucose 5.6, CaCl_2_ 2.5. The tissues were maintained at 37°C and continually gassed with 5% CO_2_ in O_2_. A passive tension of 0.5 g was applied to mouse and guinea pig tracheas and dog bronchi and 1 g was applied for human bronchial preparations. Tension was maintained for a 30–60 minutes equilibration period until it achieved a steady state. Tissues were then contracted with methacholine (10^−5^ M), a muscarinic agonist, or histamine (10^−5^ M) (for guinea pig tracheas), cumulative concentrations of ASM-024 (10^−7^ to 10^−3^ M) were added and changes of tension recorded. The results are expressed as a percentage of maximal contraction.

To assess the potential protective effect on smooth muscle contraction, isolated mouse tracheas were first exposed to 10^−5^ M methacholine and the reference contraction was recorded. Once a plateau was reached, the tracheas were washed and re-equilibrated for 30 minutes and then treated for 10 minutes with ASM-024 (10^−6^ to 10^−3^ M) or vehicle, and contractile response to increasing concentrations of methacholine (10^−8^ to 10^−5^ M) were recorded; contraction is expressed as percentage of the maximal reference tension induced by 10^−5^ M methacholine. In another set of experiment, ASM-024 pre-treated tracheas were washed extensively and after a period of 15 or 60 minutes were contracted with increasing concentrations of methacholine.

In some experiments, mouse tracheas were exposed to 10^−2^ M and 10^−1^ M hexamethonium, a non-selective nAChR antagonist, for 10 minutes before the addition of increasing concentrations of ASM-024.

### Statistical Analysis

Data in the text and figure legends are expressed as mean ± SD. For isometric studies, sample size (n values) equal the number of animals from which tracheas and bronchi were taken. EC_50_ and ED_250_ values were estimated by linear regression of individual concentration-responses curves using XLfit^TM^ (IDBS). Statistical significance of differences between means was determined by one-way ANOVA followed by Tukey test. For AR and isometric experiments, data were analyzed using a repeated mixed model. The multivariate normality assumptions were verified with the Shapiro-Wilk test after a Cholesky factorization. The Brown and Forsythe's variation of Levene's test statistic was used to verify the homogeneity of variances (statistical package SAS, version 9.1.3, SAS Institute Inc., Cary, NC, U.S.A.). The results were considered significant with p-values < 0.05.

## Results

### *In Vivo* Anti-inflammatory Effects in Allergen-Sensitized Mice

**Figure 1:**
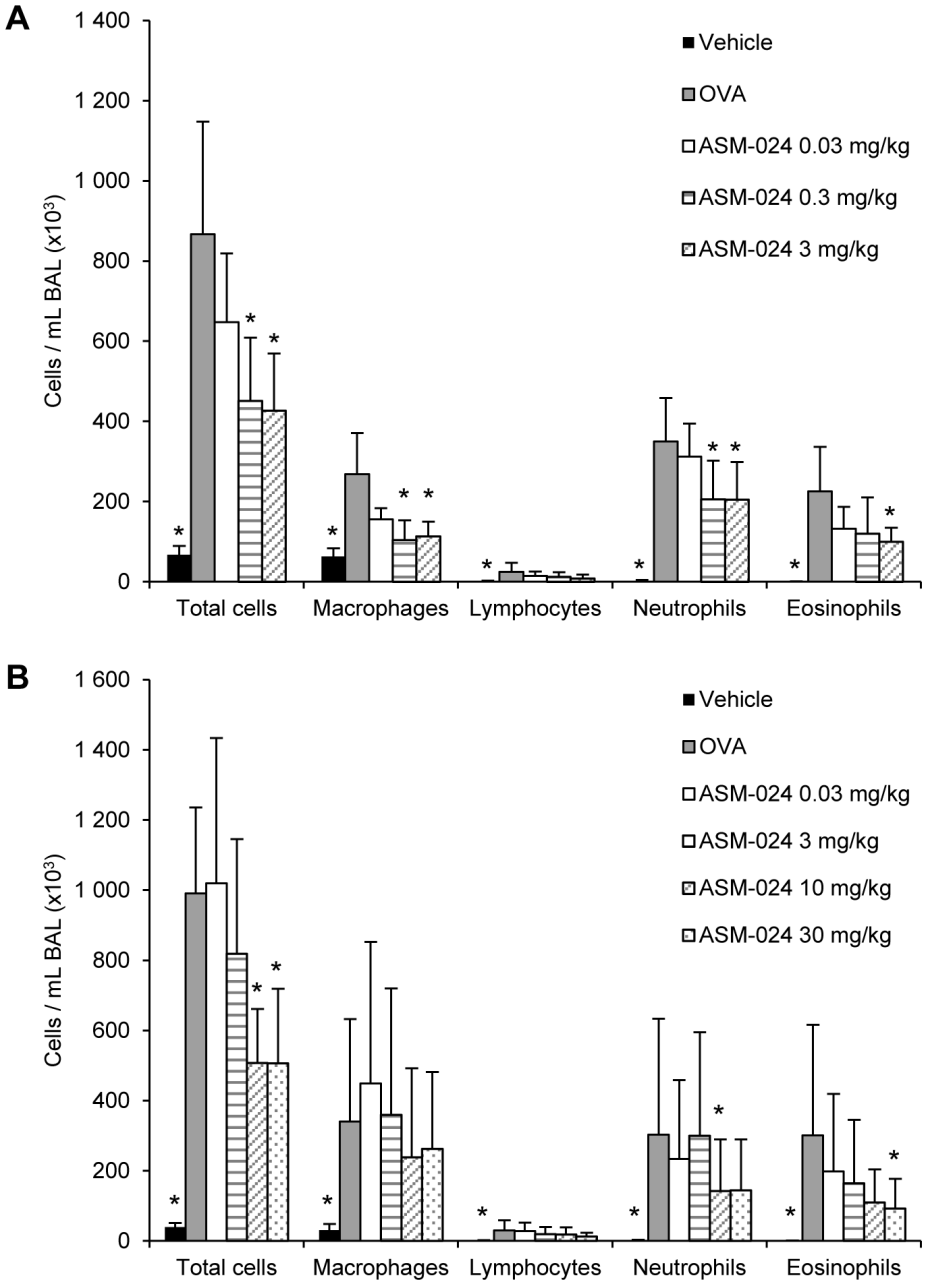
Effect of ASM-024 on cellular inflammation. ASM-024 was administered by (A) nebulization of a solution and (B) orally to OVA-sensitized mice. A dose-related inhibition of inflammation is observed, no further inhibitory effect observed at a dose higher than 10 mg/kg p.o.; Values are mean ± SD. * Significantly different from vehicle control groups; p≤0.001; n = 8–9; ED_50_ p.o. = 3.55 mg/kg; ED_50_ inhalation = 0.03 mg/kg.

Mice that received ASM-024 at doses up to 30 mg/kg p.o. showed no clinical evidence of side effects. The pharmacological effects of ASM-024 are presented in figures 1a and b. ASM-024, given by oral or nebulisation to OVA-sensitized mice decreased lung inflammation as demonstrated by a dose dependent decrease in the number of inflammatory cells recovered by BAL. Although the number of cells did not decrease to the levels found in control non-sensitized animals, the decrease was dose proportional and highly significant (p < 0.002) when compared to OVA-sensitized mice which did not receive ASM-024. BAL cell differential counts showed that all cell populations, including eosinophils, were decreased and that the relative percentages remained similar.

### *In Vivo* Airway Responsiveness in Allergen-Sensitized Mice

The methacholine ED_250_ values indicated that airway responsiveness (AR) to methacholine was significantly decreased following administration of ASM-024 by inhalation at doses of 1.5 and 3 mg/kg but not at 0.3 mg/kg. A statistically significant attenuation of AR was also observed after intranasal instillation of ASM-024 at a dose of 3 mg/kg (Table 1).

**Table 1:** Effective dose of methacholine (mg/kg) required to induce a 250% increase of airway resistance.

Group	Mean ± SD ED_250_
Control naive mice	0.234±0.043 *
OVA_Vehicle	0.089±0.031
OVA_ ASM-024 0.3 mg/kg^a^	0.111±0.027
OVA_ ASM-024 1.5 mg/kg^a^	0.145±0.032*
OVA_ ASM-024 3.0 mg/kg^a^	0.128±0.037*
OVA_ ASM-024 3.0 mg/kg^b^	0.159±0.043*

a Inhalation, ^b^ Intranasal

* p<0.05 compared to vehicle-treated OVA-sensitized mice.

ASM-024 administered by intranasal or inhalation routes immediately prior to the methacholine challenge to OVA-sensitized mice attenuated airway hyper-responsiveness.

**Figure 2:**
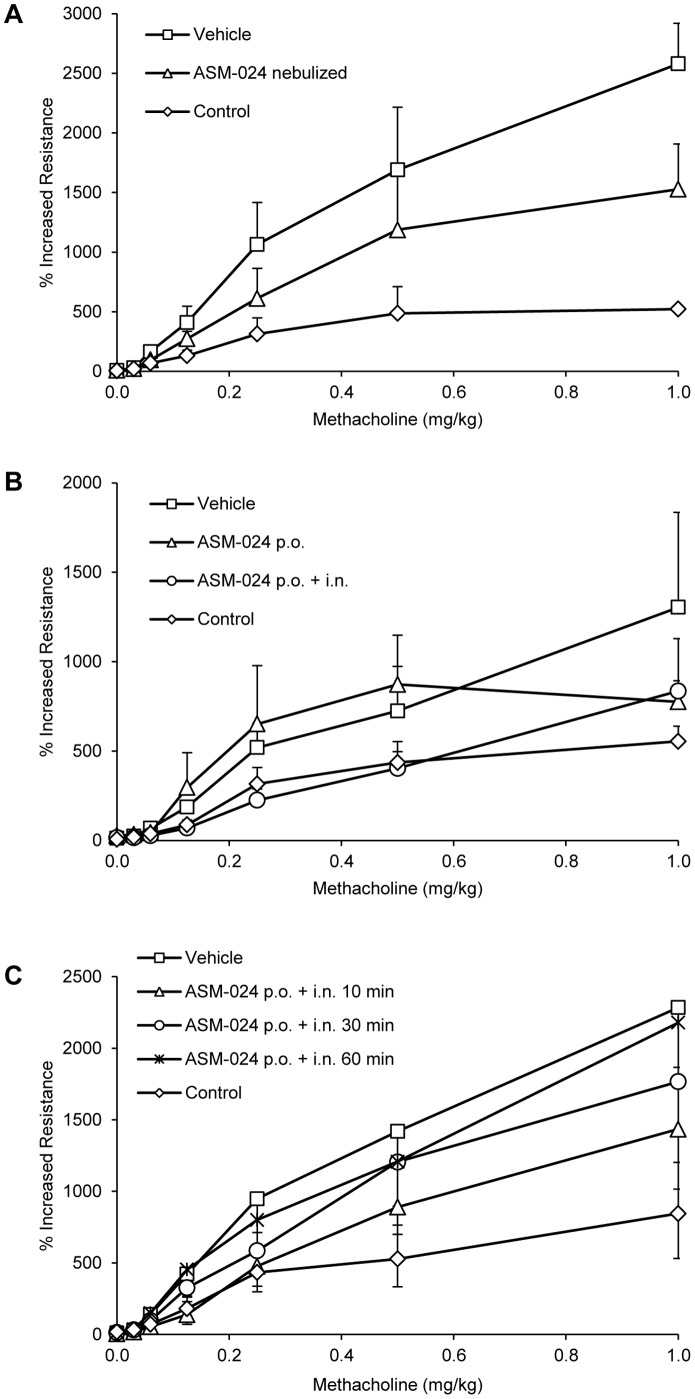
Effect of ASM-024 on lung tissue resistance in response to methacholine. ASM-024 was administered by (A) nebulization of a solution and (B and C) orally to OVA-sensitized mice. A single 5 minutes exposure to 1.5 mg/kg nebulized ASM-024 prior to the methacholine challenge significantly decreased airway resistance in OVA-sensitized mice; p<0.05 for 0.06, 0.125, 0.25 and 1 mg/kg methacholine (A). Oral administration of 4 mg/kg ASM-024 during 7 days has no significant effect on AR. An additional intranasal instillation of 4 mg/kg ASM-024, 15 minutes before the methacholine challenge reduced AR; p<0.05 for 0.06 to 0.5 mg/kg methacholine. Values are expressed as a percentage of increased resistance; mean ± SD; n = 3–8 for control, n = 6–8 each for untreated and ASM-024-treated groups (B). The local effect on airway resistance is time-dependant; maximal relaxation is observed at about 10 minutes before the methacholine challenge (C).

No significant effect on AR was observed following oral administration of 4 mg/kg during 7 days. A previous experiment where a dose of 10 mg/kg was used showed no effect on AR (data not shown). However, a significant reduction was observed when the drug was administered by intranasal instillation at a dose of 4 mg/kg, 15 minutes before the methacholine challenge (Figure 2b), and this effect was time-dependant (Figure 2c).

### *In vitro* Smooth Muscle Relaxant and Protective Effects

**Figure 3:**
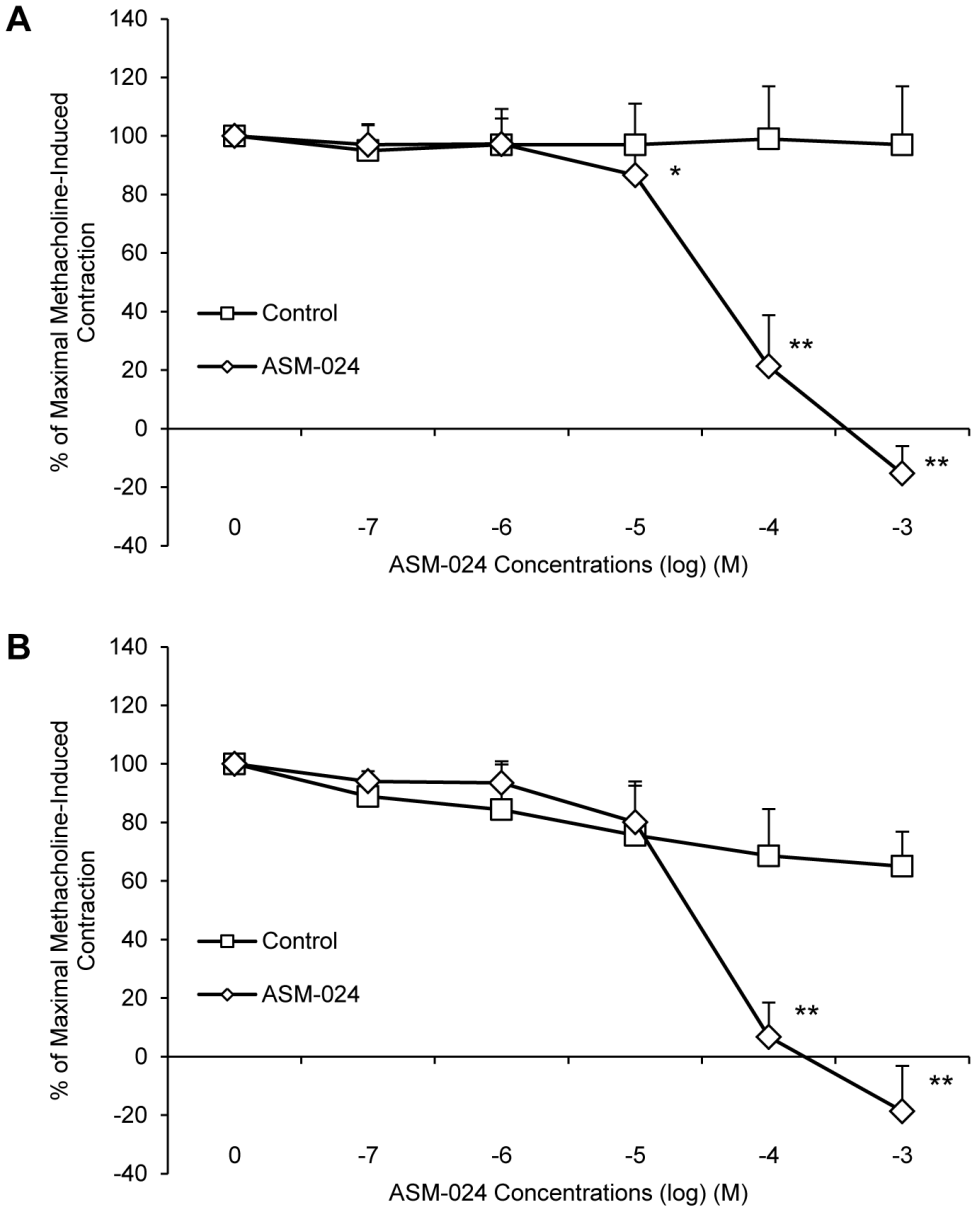
*In vitro* ASM-024-induced relaxation of methacholine-contracted (A) mouse and (B) guinea pig tracheas. Values (mean ± SD) are expressed as% of maximal methacholine-induced contraction; n = 10–18. Mouse tracheas EC_50_ = 54.15±24.15 µM, guinea pig trachea EC_50_ = 43.39±19.19 µM. * Significantly different from vehicle control, p<0.05; ** significantly different from vehicle control, p<0.0001.

ASM-024 produced a significant dose-related relaxation of methacholine-contracted mouse and guinea-pig tracheas with an overall mean EC_50_ value of 62.23±17.54 µM for mouse and 43.39±19.19 for guinea pig. Control tracheas maintained their contraction (Figures 3a and 3b).

**Figure 4:**
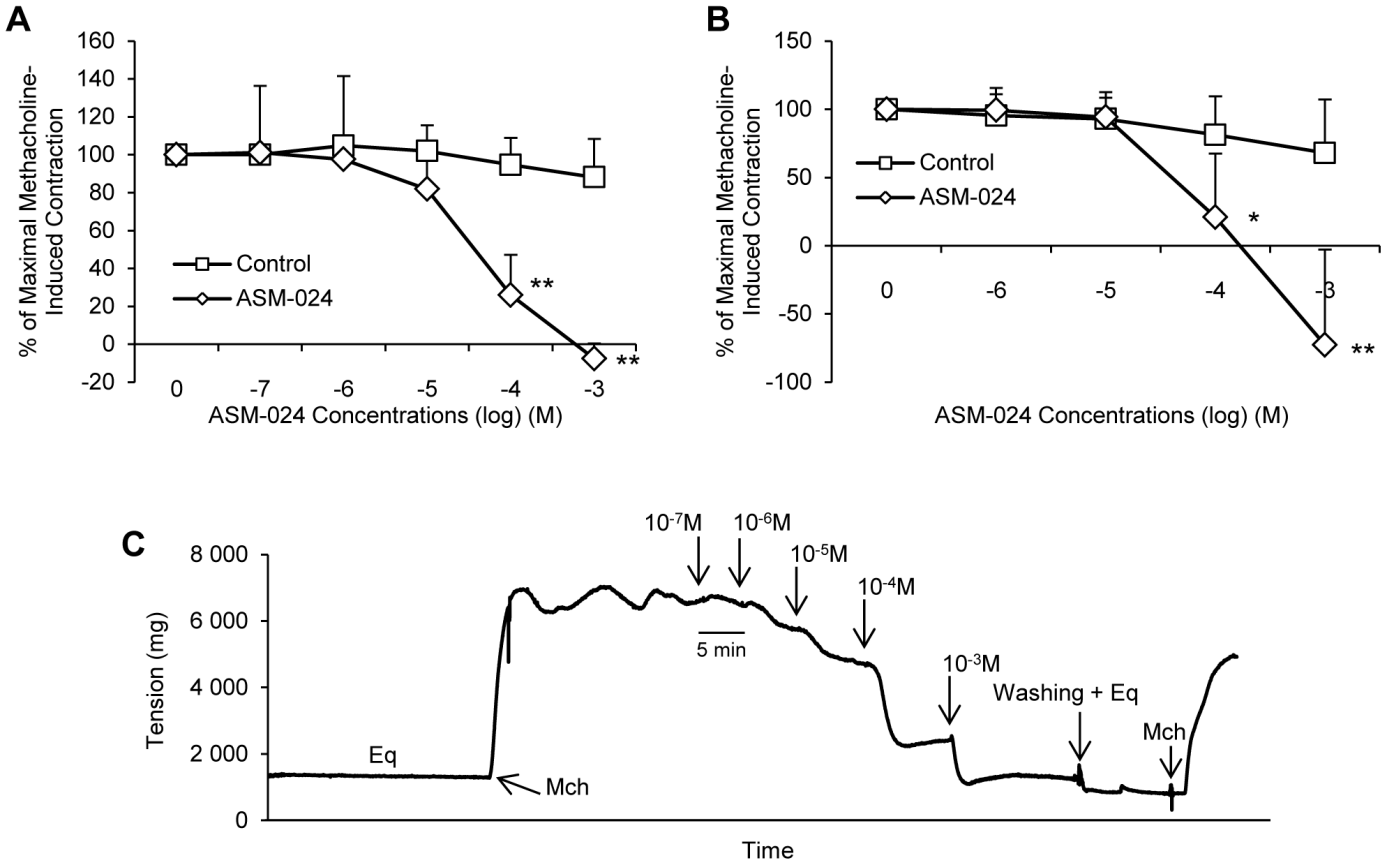
*In vitro* ASM-024-induced relaxation of methacholine-contracted (A) dog and (B) human bronchi preparations and representative tension recording depicting relaxation response of dog bronchial rings and partial reversion after washing (C). Values expressed as% of maximal methacholine-induced contraction are means ± SD; n = 8–18. * Significantly different from vehicle control, p<0.05; ** significantly different from vehicle control, p≤0.0001. Dog bronchi EC_50_ = 60.51±30.76 µM, human bronchi EC_50_ = 110.28±36.96 µM. Eq: equilibrium, MCh: methacholine.

Similar experiments were performed using dog bronchi and human bronchial preparations obtained from patients undergoing lung resection. A dose-dependent smooth muscle relaxation of dog (Figure 4a) and human (Figure 4b) pre-contracted bronchi was observed.

**Figure 5:**
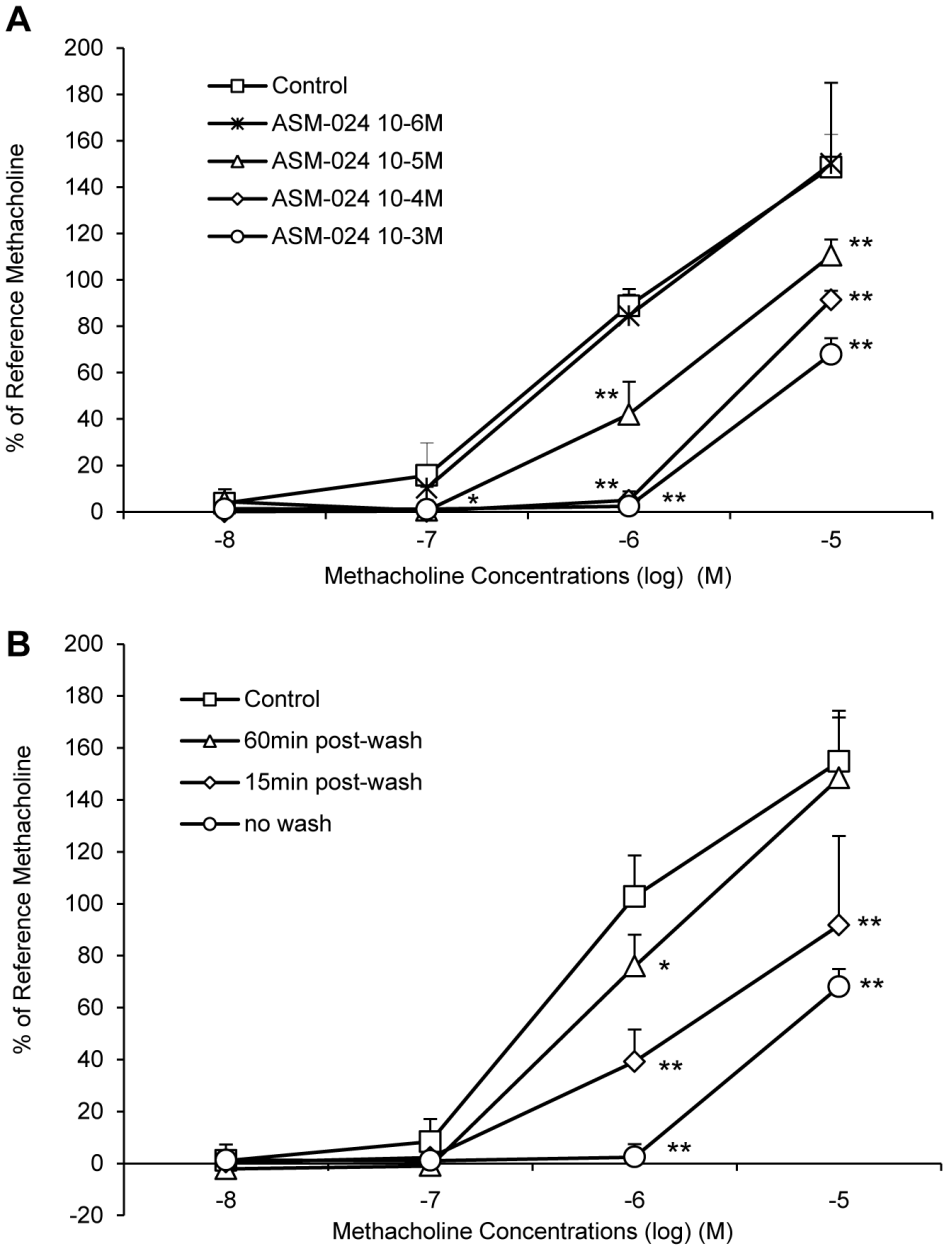
Protective effect of ASM-024 on methacholine-induced contraction of mouse tracheas. (A) A dose-related inhibition of contraction is observed. (B) A time-dependant decrease in methacholine-induced contraction is observed up to 60 minutes after ASM-024 removal from the milieu. Application of ASM-024 alone did not affect basal tension. Values expressed as% of maximal methacholine-induced contraction (10^−5^ M) recorded prior to ASM-024 treatments are means ± SD; n = 3–4.

ASM-024-induced relaxation occurs within minutes depending on the concentration. Steady state is obtained within 3 to 5 minutes and was maintained for as long as the compound was in the organ bath. It was however partially reversible as extensive washing of the tracheas followed by the addition of methacholine resulted in the return of the contraction at a magnitude lower than the initial contraction (Figure 4c). Once the relaxation effect of ASM-024 was well demonstrated, we verified if the drug could block the contraction induced by methacholine. The results of this experiment are presented in Figure 5 where it can be seen that ASM-024 at a 10^−3^ to 10^−5^ M concentration attenuated the contractile effect of methacholine (Figure 5a). This effect was transient since it had waned after ASM-024 had been removed 15 minutes prior to the addition of methacholine and almost completely disappeared after 60 minutes of the removal of ASM-024 (Figure 5b).

**Figure 6:**
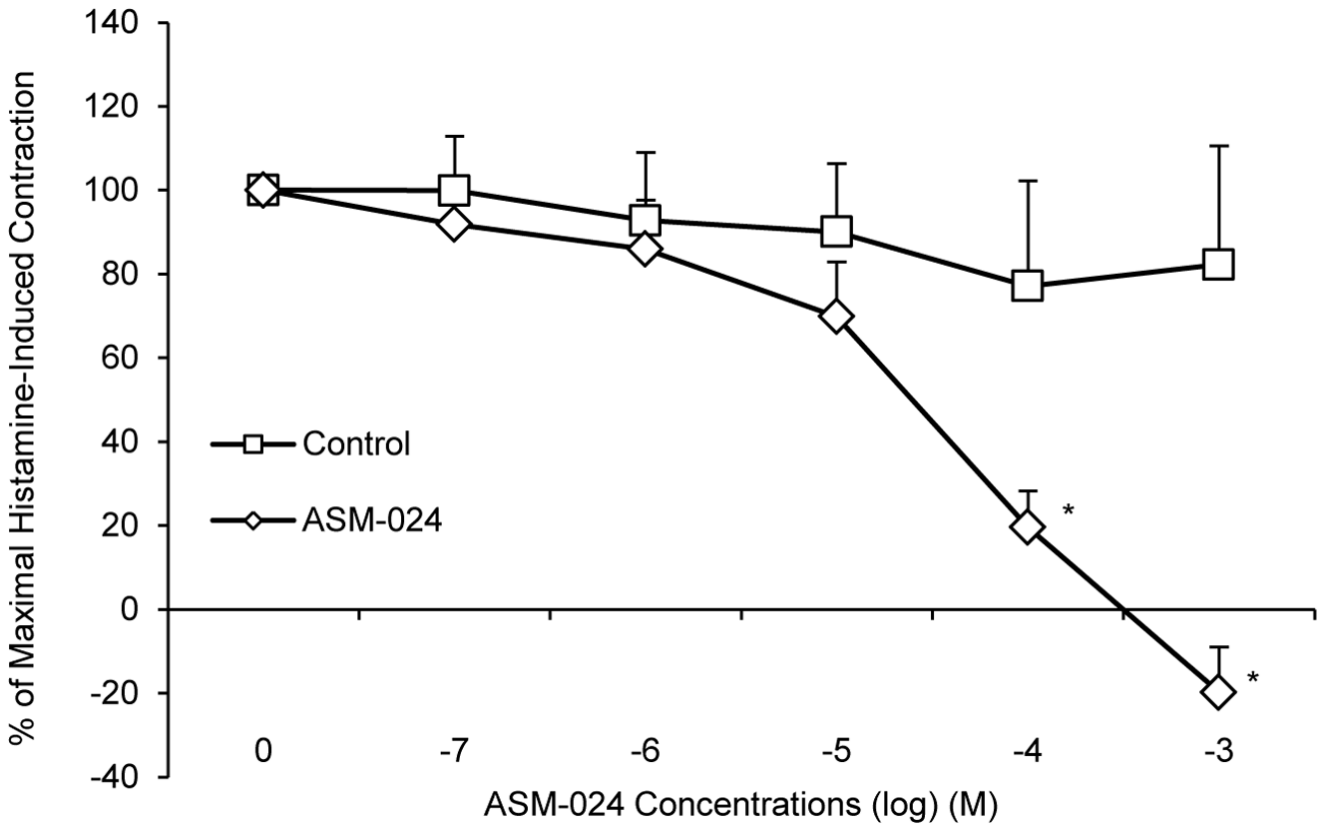
*In vitro* ASM-024-induced relaxation of histamine-contracted guinea pig tracheas. Values expressed as% of maximal histamine-induced contraction are means ± SD; n = 5–9. * Significantly different from vehicle control, p<0.001. EC_50_ = 39.06±29.10 µM.

**Figure 7:**
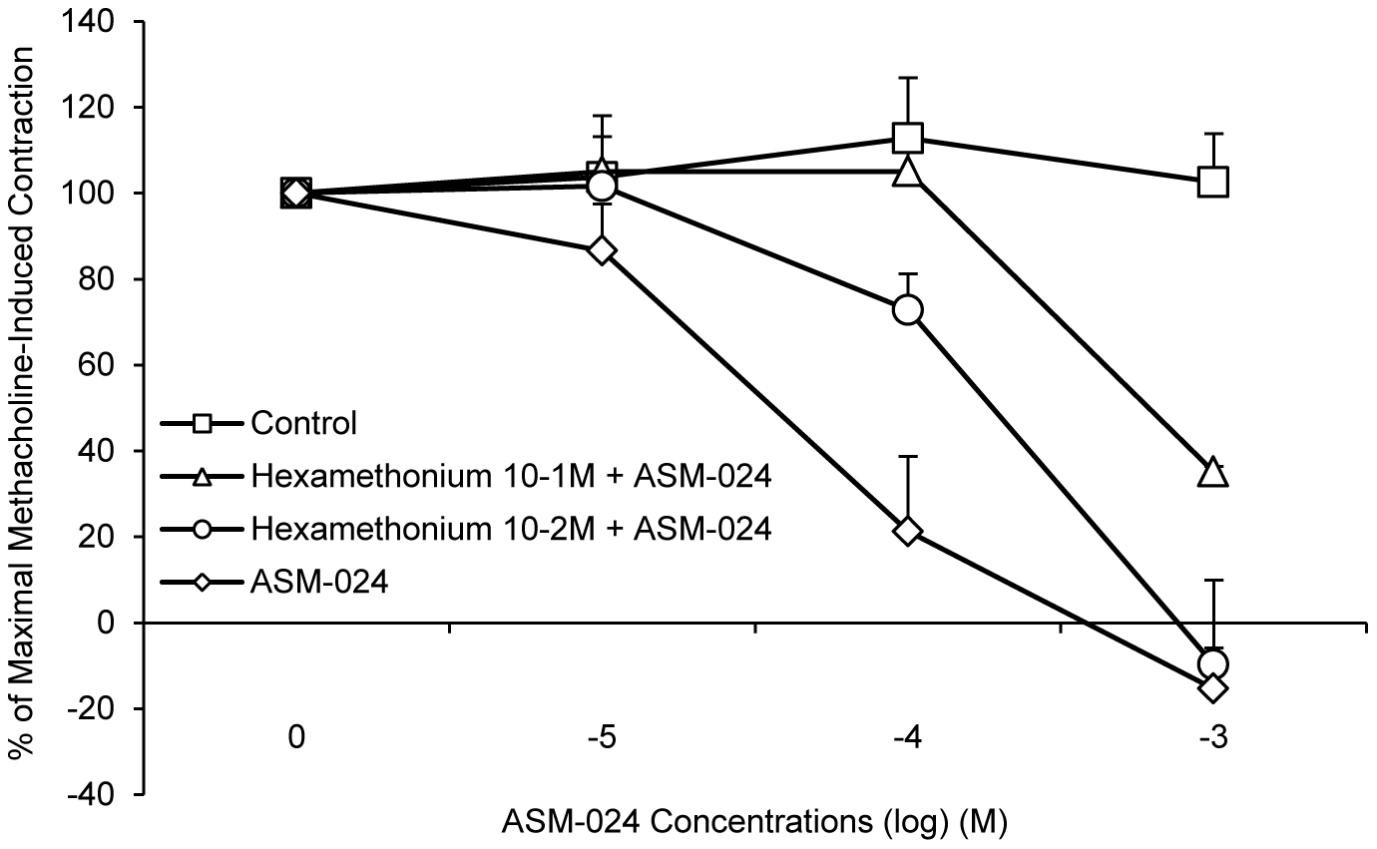
Partial reversal of ASM-024-induced relaxation. A dose-dependent shift to the right in the dose-response curve of ASM-024-induced relaxation is observed upon pre-treatment with hexamethonium, a nAChR antagonist (EC_50_ =  239.13±41.37 µM and 540.60±1.00 µM vs 62.23±17.54 µM for ASM-024). Values expressed as% of maximal methacholine-induced contraction (10^−5^ M) after addition of hexamethonium recorded prior to ASM-024 treatments are means ± SD; n = 2–4.

To evaluate whether ASM-024 is able to induce relaxation of a non-muscarinic contractile agent, its effect on histamine-induced contraction was evaluated. The experiments were conducted on guinea pig tracheas which, contrary to mouse tracheas, express histamine receptors (Figure 6). ASM-024 dose dependently reduced the contractile response to histamine (EC_50_ =  39.06±29.10 µM). The addition of hexamethonium, a subtype non-specific non-competitive nAChR antagonist, resulted in partial reversal of ASM-024-induced myorelaxation in methacholine pre-contracted mouse tracheas. The EC_50_ values were 239.13±41.37 µM and 540.60±1.00 µM for hexamethonium (10^−2^ M) + ASM-024, and hexamethonium (10^−1^ M) + ASM-024, respectively (figure 7).

## Discussion

Although this study was not designed to specifically address the toxicology profile of ASM-024, the results show that this drug can induce significant pharmarcological activities relevant to asthma at doses that showed no evidence of toxicity either in *in vivo* or *in vitro* models. In the course of the clinical development of ASM-024, a comprehensive nonclinical safety program was conducted with ASM-024 in rats and dogs including safety pharmacology, toxicology, and genotoxicity studies. Under single oral administration, ASM-024 was well tolerated at oral doses up to 320 mg/kg in rats and 120 mg/kg in dogs and up to 40 mg/kg in both rats and dogs by nebulization [35]. These doses are well above the pharmacological active doses determined in mouse models of allergic airway inflammation and hyperreactivity. ASM-024 showed no evidence of genotoxicity in the standard battery of genotoxicity studies and no evidence of producing hypersensitisation in an inhalation model in the guinea pig. Results of the studies reported here show that ASM-024 has anti-inflammatory, smooth muscle relaxant and bronchoprotective properties. Airway cellular inflammation was attenuated following ASM-024 administration both via oral and inhalation routes, indicating that the drug displays both local and systemic anti-inflammatory activity, although the doses for which a pharmacological effect is observed were higher by the oral administration. This may be due to poor oral systemic absorption and/or rapid hepatic first pass metabolism. The pharmacological effects observed with ASM-024 were comparable to those observed with DMPP [20] and ASM-002, another ASM-024 analogue (unpublished data). These effects were associated with an *in vivo* reduction of serum IgE, lung tissue inflammation and *in vitro* reduction of eosinophil migration and LTC4 release. However these latest experiments were not repeated with ASM-024.

Moreover, direct short term local administration either through intranasal treatment or by nose-only nebulization just before a methacholine challenge led to a significant reduction in airway tissue resistance which was not observed after oral or nebulized administration suggesting that ASM-024 has a direct local effect on airway smooth muscle relaxation. These results indicate that the decrease in inflammatory response is not necessarily associated with a reduction of airway hyperresponsiveness, but indicate a dual mechanism of action on cellular inflammation independent of the direct airway relaxation effect.

In isometric *in vitro* studies, ASM-024 inhibited the contractile response to methacholine and histamine in a dose-dependent and reversible manner. Of interest is that a similar smooth muscle relaxation was observed in the four species studied, including man. Not only does ASM-024 induced relaxation it also had a protective effect on methacholine-induced constriction.

Most structural and inflammatory cells present in the respiratory tract express nicotinic and/or muscarinic receptors and both types have been involved in the regulation of inflammatory responses [12], [25]. Activation of nicotinic receptors by acetylcholine and nicotinic agonists, particularly the α7 nAChR subtype [13] but also other subtypes, can limit inflammation through signal transduction pathways that do not involve ion channel activation [26]–[28]. Activation of muscarinic receptors by acetylcholine has pro-inflammatory and airway remodelling effects that are associated with COPD and asthma [15], and indeed there is now evidence that muscarinic receptor antagonists are also associated with anti-inflammatory, anti-proliferative, and anti-remodeling effects [25], [29].

The precise pharmacological target and mode of action of ASM-024 on inflammation and smooth muscle relaxation has not been fully elucidated. ASM-024 is a DMPP derivative and although it is a weak agonist, a modulatory role via nicotinic receptors is probable. Recent observations have indicated that ASM-024 may function as a "silent nicotinic receptor agonist" since alone it does not induce activation of the nicotinic ion channel but when co-applied with the positive α7 receptor allosteric modulator PNU-120596, it is able to activate the α7 receptor ion channel currents [23]. Ongoing studies are being conducted to demonstrate that ASM-024 and other similar pharmacophore(s) may act on signaling pathways independently of ion channel activation. Indeed, other signal transduction mechanisms not involving channel opening have been shown to occur via certain subtype-selective nAChRs [30]–[33].

In isometric studies, the effectiveness of ASM-024 to relax both methacholine- and histamine-induced tracheal contraction suggest that a mechanism other than specific antagonism of cholinergic muscarinic receptors is involved in the antispasmodic action. Recent observations have indicated that ASM-024 dose-dependently inhibited intracellular Ca^2+^ increase on cultured tracheo-bronchial human smooth muscle cells stimulated with histamine (unpublished data). The precise mechanism of ASM-024 effect on intracellular calcium decrease has yet to be defined: Ca^2+^ influx through other membrane Ca^2+^ channels, Ca^2+^ mobilisation, Ca^2+^ reuptake and/or Ca^2+^ efflux are other important pathways being investigated. ASM-024 may have a different pharmacological activity than currently-available compounds for the treatment of asthma and COPD. Since the epithelium was not removed, an indirect effect through epithelium derived factors cannot be ruled out. However the rapidity of the relaxation may suggest a direct effect at the airway smooth muscle level. Nicotinic receptors are expressed on airway smooth muscle [21], [30] but their role on these cells is unknown. In a previous study using the nicotinic agonist DMPP [21], the relaxant effect was partially reversed with hexamethonium a non-selective, nAChR antagonist, suggesting a potential role of nicotinic receptors in the relaxation response, similar results were observed with ASM-024. nAChRs are permeable to Na^+^ and K^+^ and to a lesser extent to Ca^2+^ so a direct modulatory role of cation conductance by ASM-024 through these receptors is possible.

Although the concentrations used to achieve pharmacological responses *in vitro* were relatively high, the *in vivo* doses were within well tolerated range and suggest that the amount of drug that would be required to achieve clinically relevant pharmacological effects would not preclude its potential use in humans to treat asthma. One explanation for this disconnect between *in vitro* and *in vivo* data may be related to the rapid desensitization of the nicotinic receptor upon binding of the ligand, essentially resulting in the need for continuous exposure to higher concentrations in order to achieve a pharmacological effect *in vitro* whereas *in vivo* recovery from the desensitized state may occur more rapidly [34].

Further studies are planned to define the precise mechanisms of action for ASM-024. ASM-024 appears to be a modulator of cholinergic receptor function. Its action seems to be multi-mediated as it has demonstrated a novel three-fold mechanism of action of i) non-competitive inhibition nicotinic receptor ion channel function, ii) potential orthosteric modulation of α7-mediated signal transduction via non-conducting states and iii) blockade of muscarinic activity by a mechanism independent of a direct specific antagonism of cholinergic muscarinic receptors.

Overall, this compound displays anti-inflammatory, smooth muscle relaxant and bronchoprotective properties, with a different pharmacological profile than currently available compounds for the treatment of asthma and COPD and may represent an interesting and new approach to treat these pulmonary diseases.
